# Phenolic Compounds in Exercise Physiology: Dual Role in Oxidative Stress and Recovery Adaptation

**DOI:** 10.1002/fsn3.70714

**Published:** 2025-07-28

**Authors:** Kübra Özdemir, Yeliz Demir

**Affiliations:** ^1^ Faculty of Sports Sciences, Physical Education and Sports Teaching Department Atatürk University Erzurum Türkiye; ^2^ Nihat Delibalta Göle Vocational High School, Department of Pharmacy Services Ardahan University Ardahan Türkiye; ^3^ Faculty of Science, Department of Chemistry Atatürk University Erzurum Türkiye

**Keywords:** exercise, oxidative stress, phenolic compound, reactive oxygen species

## Abstract

Exercise‐induced oxidative stress results from the overproduction of reactive oxygen and nitrogen species (ROS/RNS) during intense physical activity, potentially impairing muscle function and recovery. Phenolic compounds, abundant in plant‐based foods, are known for their potent antioxidant properties and may modulate redox homeostasis in athletes. This review critically examines the dual role of phenolic compounds in exercise physiology, highlighting both their protective antioxidant effects and the risks of excessive intake that may disrupt adaptive responses. We provide an overview of their molecular mechanisms, dose‐dependent outcomes, and bioavailability issues, alongside evidence from animal and human studies. Notably, excessive antioxidant supplementation may interfere with beneficial exercise‐induced adaptations, including mitochondrial biogenesis. The review also emphasizes the need for personalized antioxidant strategies based on individual training status, exercise intensity, and metabolic variability. Future research should address long‐term effects, optimal dosing, and the interaction of phenolic compounds with other dietary antioxidants. Our findings aim to inform evidence‐based recommendations for integrating phenolic compounds into exercise recovery and performance strategies.

## Graphical abstract

Exercise increases mitochondrial activity and reactive oxygen species (ROS) production, which can lead to oxidative stress. However, phenolic compounds act as potent antioxidants, counteracting ROS and thereby mitigating oxidative stress. This graphical abstract illustrates the dual role of exercise and dietary antioxidants in the regulation of redox balance.

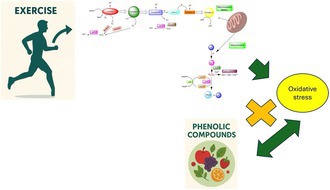

## 1 Introduction

Reactive oxygen species (ROS) and reactive nitrogen species (RNS) are constantly generated in aerobic organisms, and their production increases substantially during physical exercise (Jeppesen et al. 2024). Although excessive accumulation of these reactive molecules may lead to oxidative stress, impairing muscle function and delaying recovery, moderate levels are essential for cellular signaling processes involved in physiological adaptation (Qaisar et al. 2016). This duality forms the basis for ongoing discussions on the role of antioxidant interventions in exercise physiology. Among dietary antioxidants, phenolic compounds have garnered attention due to their high abundance in plant‐based foods and their potent redox‐modulating properties (I. Gülçin 2006a, 2006b; İ. Gülçin 2010). These secondary metabolites, including flavonoids, phenolic acids, and stilbenes, are known to scavenge free radicals, chelate pro‐oxidant metal ions, and upregulate endogenous antioxidant enzymes such as superoxide dismutase (SOD), catalase (CAT), and glutathione peroxidase (GPx) (İ. Gülçin 2011; Topal et al. 2016). Mechanistically, they act at the molecular level by activating the nuclear factor erythroid 2‐related factor 2 (Nrf2)/Antioxidant Response Element (ARE) signaling pathway to increase the expression of key antioxidant enzymes (Krajka‐Kuźniak et al. 2013), inhibiting inflammatory mediators via Nuclear Factor kappa‐light‐chain‐enhancer of activated B cells (NF‐κB) suppression, and enhancing mitochondrial biogenesis through Peroxisome Proliferator‐Activated Receptor Gamma Coactivator 1‐alpha (PGC‐1α) activation (Yang et al. 2017). These compounds therefore support exercise adaptation by reducing oxidative damage while preserving the beneficial signaling roles of ROS. Consequently, phenolic compounds are widely used in nutritional strategies aimed at reducing exercise‐induced oxidative damage and promoting recovery (Nanavati et al. 2022). However, recent evidence highlights that the effects of phenolic compounds are not uniformly beneficial. High doses or chronic supplementation may suppress beneficial ROS‐mediated signaling pathways that drive adaptations to training, such as mitochondrial biogenesis, angiogenesis, and muscle remodeling. This paradox underscores the complexity of antioxidant interventions in physically active individuals, particularly when phenolic compounds are consumed in pharmacological quantities rather than as part of a balanced diet (Wang, Yang, et al. 2023; Wang, Zhao, et al. 2023).

Physical exercise is known to modulate oxidative stress in a dose‐ and intensity‐dependent manner. Acute high‐intensity training increases ROS and RNS levels sharply, potentially causing lipid peroxidation and inflammation (Freitas et al. 2018). In contrast, chronic moderate‐intensity exercise enhances the body's antioxidant defenses through hormetic mechanisms. The interplay between exercise intensity, redox status, and dietary intake thus warrants detailed examination, especially in light of individual variability in metabolic responses, gut microbiota, genetic polymorphisms, and training level. Although several studies have investigated the antioxidant roles of phenolic compounds in exercise settings, the majority focus on acute effects or single‐outcome measures, often neglecting long‐term adaptations and potential pro‐oxidant risks (Kruk et al. 2022; Zhang et al. 2025). Moreover, many reviews fail to contextualize findings within exercise modality, training load, or phenolic subclass differences. A critical synthesis of the literature that integrates molecular mechanisms with applied performance outcomes remains lacking.

This review addresses this gap by systematically analyzing the dual role of phenolic compounds in exercise‐induced oxidative stress and recovery adaptation. We provide an overview of the current evidence from animal and human studies, highlighting both the benefits and potential risks of phenolic intake in athletic and recreational populations. Emphasis is placed on the molecular pathways involved, dose–response relationships, and the long‐term consequences of supplementation. Through this synthesis, we aim to inform the development of personalized antioxidant strategies that balance redox homeostasis without compromising the physiological adaptations essential for performance and health.

## 2 Basic Mechanisms of Oxidative Damage

### 2.1 Free Radicals

Oxidation–reduction reactions are fundamental to cellular metabolism and redox regulation. In biological systems, reductants and oxidants are often referred to as pro‐oxidants and antioxidants, and their balance defines the redox state of the cell, which significantly impacts metabolic activity and signal transduction pathways (Knaus 2020). Molecular oxygen (O_2_), a biradical in its ground state, readily participates in redox reactions, producing highly ROS such as hydroxyl radical (^·^OH), superoxide (O_2_^·−^), hydrogen peroxide (H_2_O_2_), and peroxyl radicals. Free radicals are chemically unstable species characterized by unpaired electrons and include both ROS and reactive nitrogen species (RNS) (Bingol et al. 2021; Zaric et al. 2023). Among these, the ^·^OH is the most reactive and damaging due to its high reactivity and extremely short half‐life (Halliwell et al. 2021).

### 2.2 Nitric Oxide and Peroxynitrite

**FIGURE 1:**
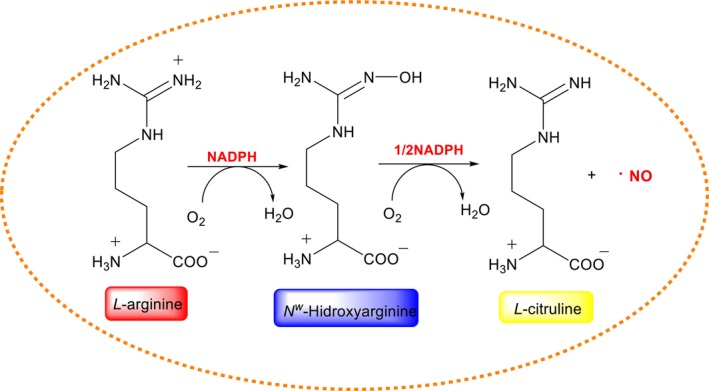
Endogenous nitric oxide (NO) synthesis by nitric oxide synthase (NOS) isoforms and its physiological relevance in cellular signaling, vascular tone regulation, and exercise‐related oxidative responses.

**FIGURE 2:**
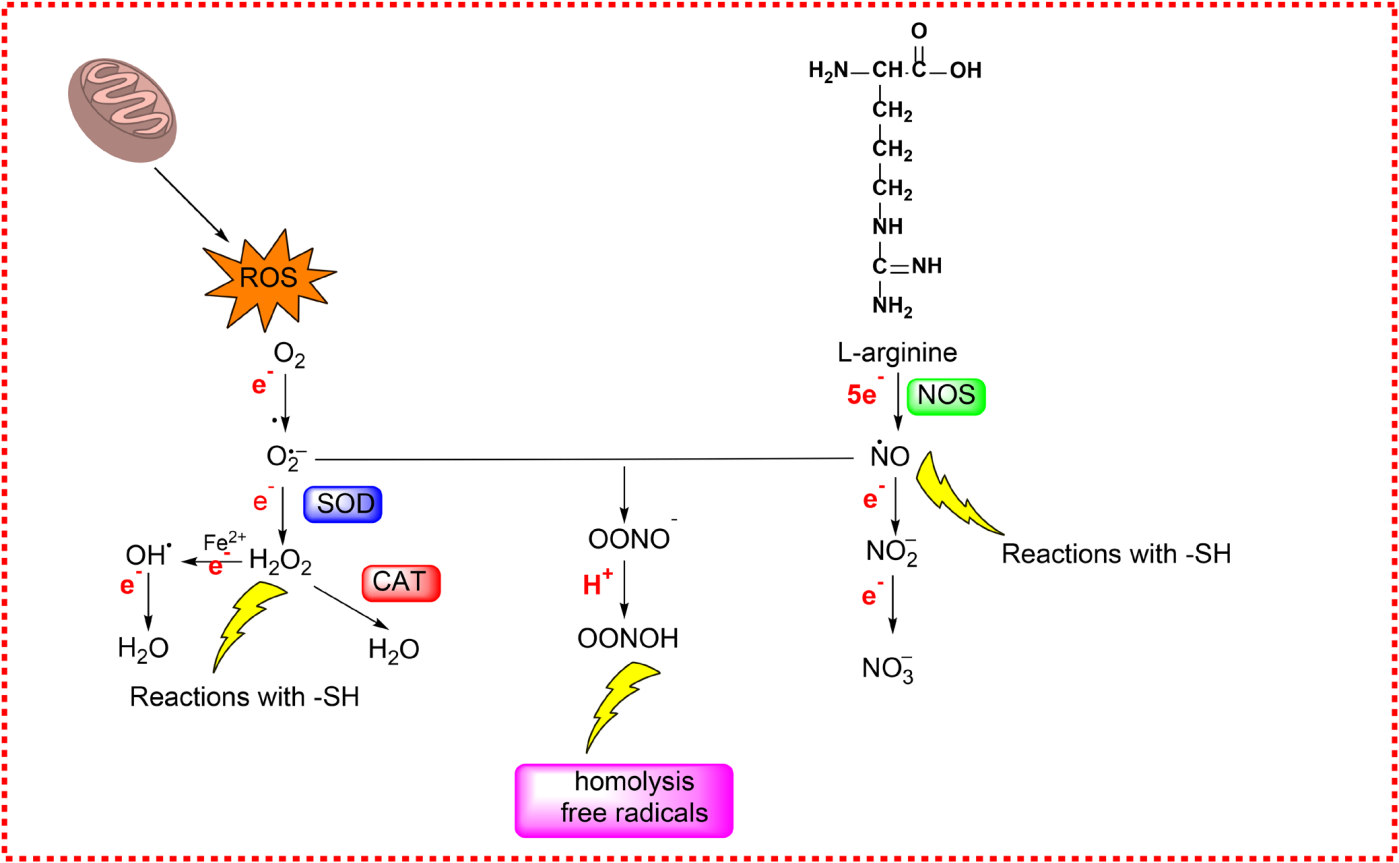
Conversion and interaction pathways of nitric oxide (^·^NO) and peroxynitrite (ONOO^−^) during exercise, highlighting their roles in oxidative and nitrosative stress, mitochondrial dysfunction, and post‐translational protein modifications.

Nitric oxide (^·^NO), a free radical produced by nitric oxide synthases (NOS), acts as a crucial signaling molecule in vasodilation and neurotransmission. ^·^NO synthesis involves oxidation of l‐arginine in the presence of NADPH and cofactors such as tetrahydrobiopterin (Figure 1) (Gonzalez et al. 2023). Under pathological or inflammatory conditions, ^·^NO reacts with O_2_^·−^ to form peroxynitrite (ONOO^−^), a potent oxidant capable of damaging DNA, lipids, and proteins (Prolo et al. 2024). Peroxynitrite generation occurs rapidly in immune and vascular cells and contributes to oxidative damage, especially when NO levels exceed the neutralizing capacity of antioxidant enzymes like SOD (Figure 2) (Fotiou et al. 2009; Su and Groves 2010).

### 2.3 Mitochondrial ROS Production

**FIGURE 3:**
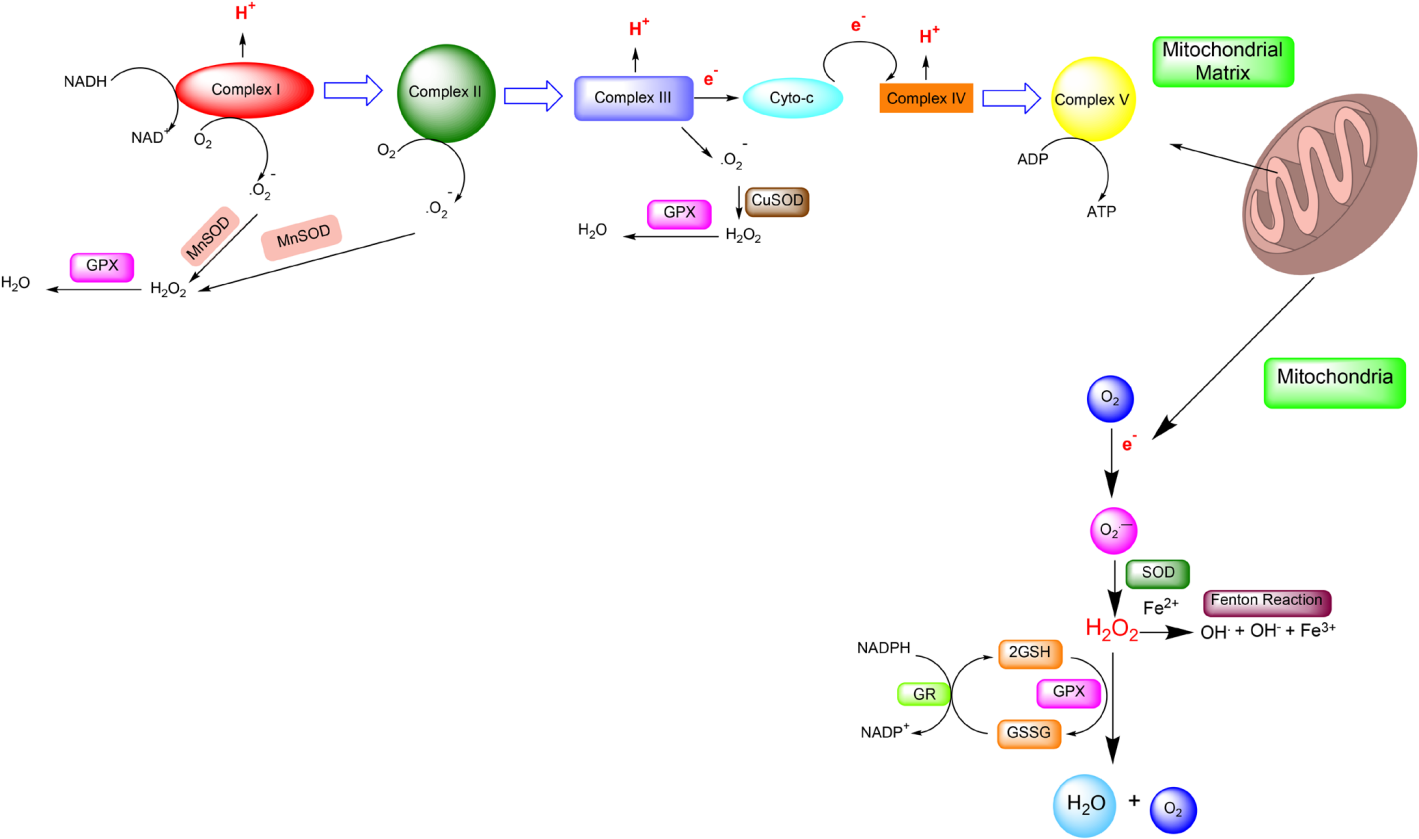
Cellular mechanisms of hydrogen peroxide (H_2_O_2_) formation: Superoxide anion (O_2_^·−^) generated via mitochondrial electron leakage, NADPH oxidase, or xanthine oxidase is dismutated by superoxide dismutase (SOD) into H_2_O_2_, which acts as a signaling molecule or contributes to oxidative damage depending on its concentration and localization.

The mitochondrial electron transport chain (ETC) is a primary endogenous source of ROS, particularly during increased energy demand such as exercise. Electrons leaking from complexes I–III can partially reduce oxygen to form superoxide, which is dismutated by SOD into H_2_O_2_ (Figure 3) (Chenna et al. 2022; Liu et al. 2023). When mitochondrial membrane permeability increases under stress, H_2_O_2_ can leak into the cytosol, initiating inflammatory signaling cascades and oxidative damage (He et al. 2017).

### 2.4 Enzymatic Sources of ROS

**FIGURE 4:**
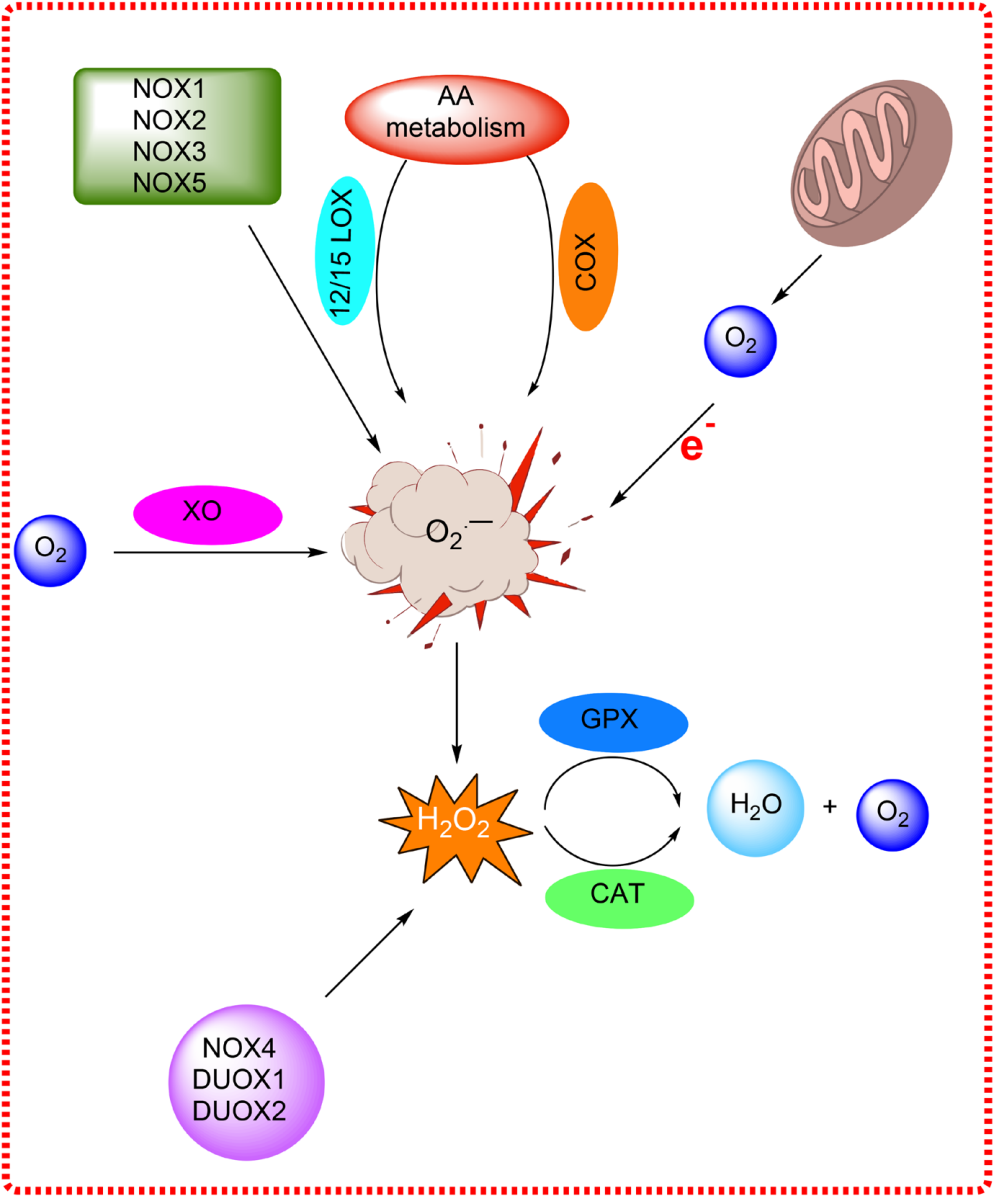
Mechanisms of superoxide radical anion formation.

In addition to mitochondria, several enzymes contribute to ROS production. These include nicotinamide adenine dinucleotide phosphate oxidases (NOXs), xanthine oxidase (XO), cyclooxygenases (COX), and lipoxygenases (LOXs). NOX enzymes transfer electrons from NADPH to oxygen, generating either superoxide or H_2_O_2_. Notably, NOX2 is activated during immune respiratory bursts, while NOX4 and DUOX produce H_2_O_2_ under basal conditions (Włodarski et al. 2020; Vermot et al. 2021). LOXs oxidize polyunsaturated fatty acids, particularly arachidonic acid, producing hydroperoxides and leukotrienes, which can further activate NADPH oxidases to amplify ROS production (Figure 4) (Kulkarni et al. 2021; Zheng et al. 2020). XO, COX, and other oxidases generate superoxide via single‐electron transfers, contributing to oxidative load during stress or intense exercise (Bortolotti et al. 2021).

### 2.5 Hydrogen Peroxide and Hydroxyl Radical Generation

Superoxide radicals generated in various pathways are readily converted into hydrogen peroxide via dismutation reactions. H_2_O_2_, although less reactive than ^·^OH, can participate in Fenton reactions in the presence of transition metals, producing highly reactive hydroxyl radicals (Liu et al. 2021). These secondary reactions amplify cellular oxidative stress and contribute to macromolecular damage if not neutralized by SODs, CAT, GPxs.

## 3 Control of ROS

Cellular ROS levels are regulated by the dynamic balance between production and elimination. According to the redox‐optimized ROS balance theory, ROS concentrations are minimized at an intermediate redox potential, neither too oxidized nor too reduced, ensuring metabolic efficiency (Nickel et al. 2014). Deviations from this balance lead to increased ROS levels, either due to excessive generation or impaired scavenging. This regulation is tightly associated with mitochondrial NADH levels and the redox states of glutathione (GSH) and NADPH, which are essential cofactors for antioxidant enzymes (George and Abrahamse 2020; Yang et al. 2021).

### 3.1 Antioxidant Enzymes

Key enzymatic antioxidants include SODs, CAT, GPxs, and the thioredoxin (Trx) system. These enzymes play critical roles in neutralizing ROS and are involved in various physiological and pathological processes (Yesilkent and Ceylan 2022; Kizir et al. 2023). SODs convert O_2_^·−^ into H_2_O_2_. SOD1 (cytosolic), SOD2 (mitochondrial), and SOD3 (extracellular) are the major isoforms. SOD1, the most abundant, also participates in transcriptional regulation and has been implicated in neurodegenerative diseases and cardiovascular conditions (Trist et al. 2021; Alateyah et al. 2022; Wang, Yang, et al. 2023; Wang, Zhao, et al. 2023).

**FIGURE 5:**
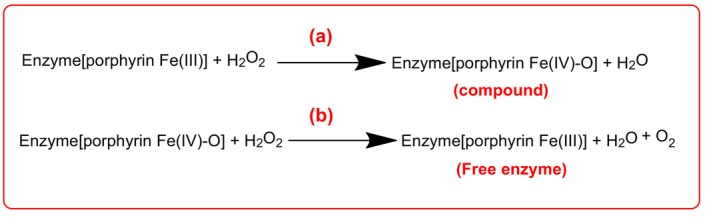
Steps in CAT reaction: (a) First step; (b) second step.

GPxs use GSH to reduce H_2_O_2_ and lipid peroxides. Their isoforms are localized to specific compartments: GPx1 (cytosol, mitochondria), GPx2 (nucleus), GPx3 (plasma), and GPx4 (membranes). They modulate signaling pathways, including insulin sensitivity and apoptosis regulation (Brigelius‐Flohé and Maiorino 2013; He et al. 2017). CAT decomposes H_2_O_2_ into water and oxygen via a two‐step mechanism involving the formation of Compound I (Figure 5a) and its reduction (Figure 5b) (Yamazaki and Piette 1990; Nandi et al. 2019).

**FIGURE 6:**
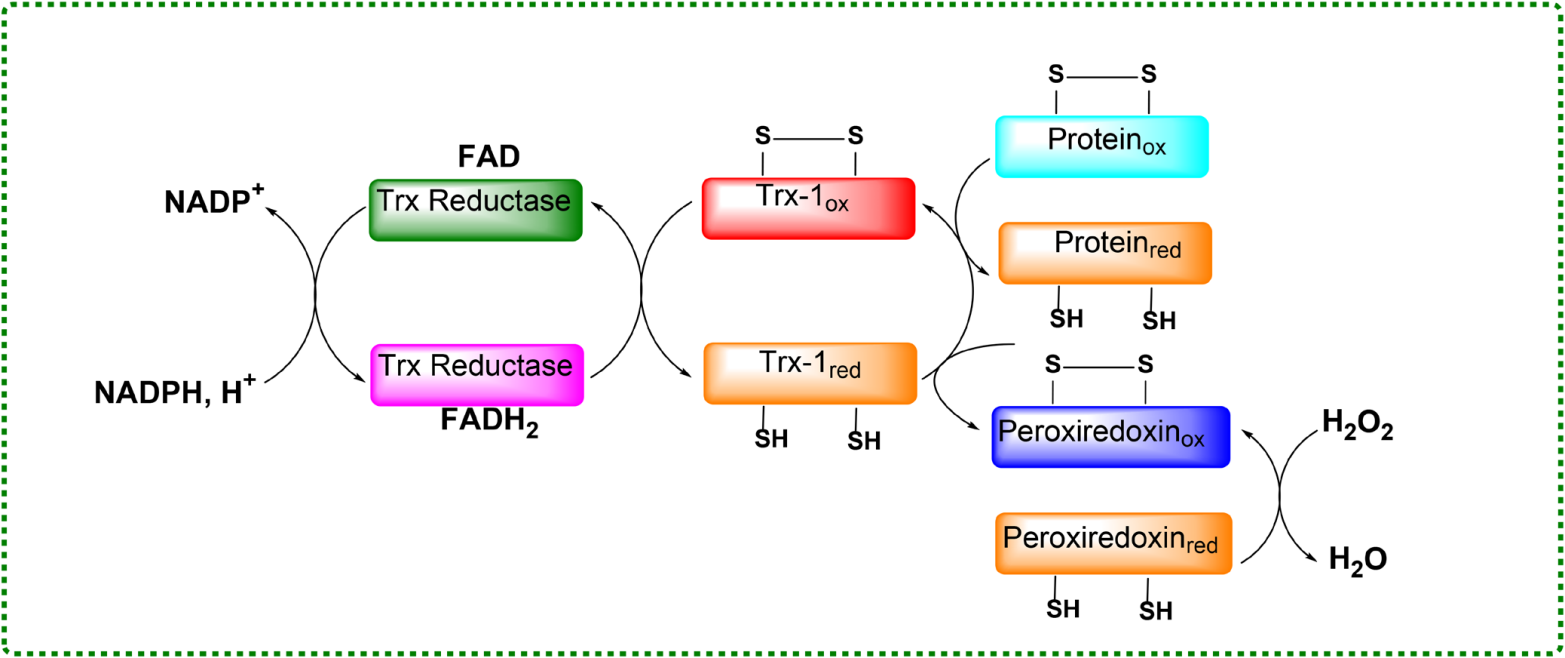
Schematic representation of the thioredoxin (Trx) system and its catalytic redox mechanism. Trx, reduced by NADPH‐dependent thioredoxin reductase (TrxR), participates in cellular redox regulation by reducing disulfide bonds in target proteins. This system plays a central role in maintaining redox homeostasis, regulating apoptosis, and modulating antioxidant responses under oxidative stress.

The Trx system includes Trx1/Trx2 and thioredoxin reductase (TrxR), maintaining redox homeostasis through NADPH‐dependent reduction (Figure 6). Trx influences apoptosis, transcription, and protein repair, and is upregulated during oxidative stress, often linked to cancer and inflammation (Bjørklund et al. 2021; Kalın et al. 2022; Günaydın et al. 2023).

### 3.2 Nonenzymatic Antioxidants

**FIGURE 7:**
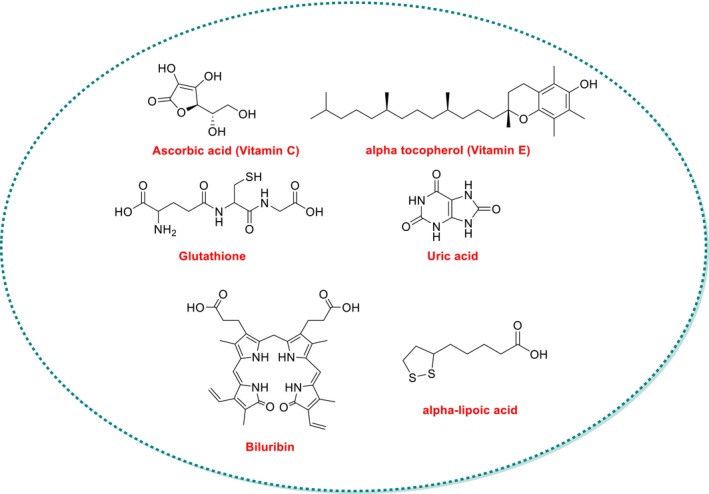
Chemical structures and physiological roles of major nonenzymatic antioxidants: Vitamin C, vitamin E, glutathione (GSH), uric acid, bilirubin, and alpha‐lipoic acid (ALA). These molecules contribute to redox homeostasis by directly scavenging ROS, regenerating other antioxidants, and modulating cellular antioxidant responses, especially during exercise‐induced oxidative stress.

Nonenzymatic antioxidants complement enzymatic systems and include vitamins C and E, GSH, bilirubin, uric acid, and alpha‐lipoic acid (Figure 7). GSH, a tripeptide, is abundant in mammalian cells and neutralizes ROS directly. It cycles between reduced (GSH) and oxidized (GSSG) states via NADPH‐dependent GSH reductase, playing a role in apoptosis prevention and redox signaling (Aoyama 2021; Güller 2021; Chatterji et al. 2022).

**FIGURE 8:**
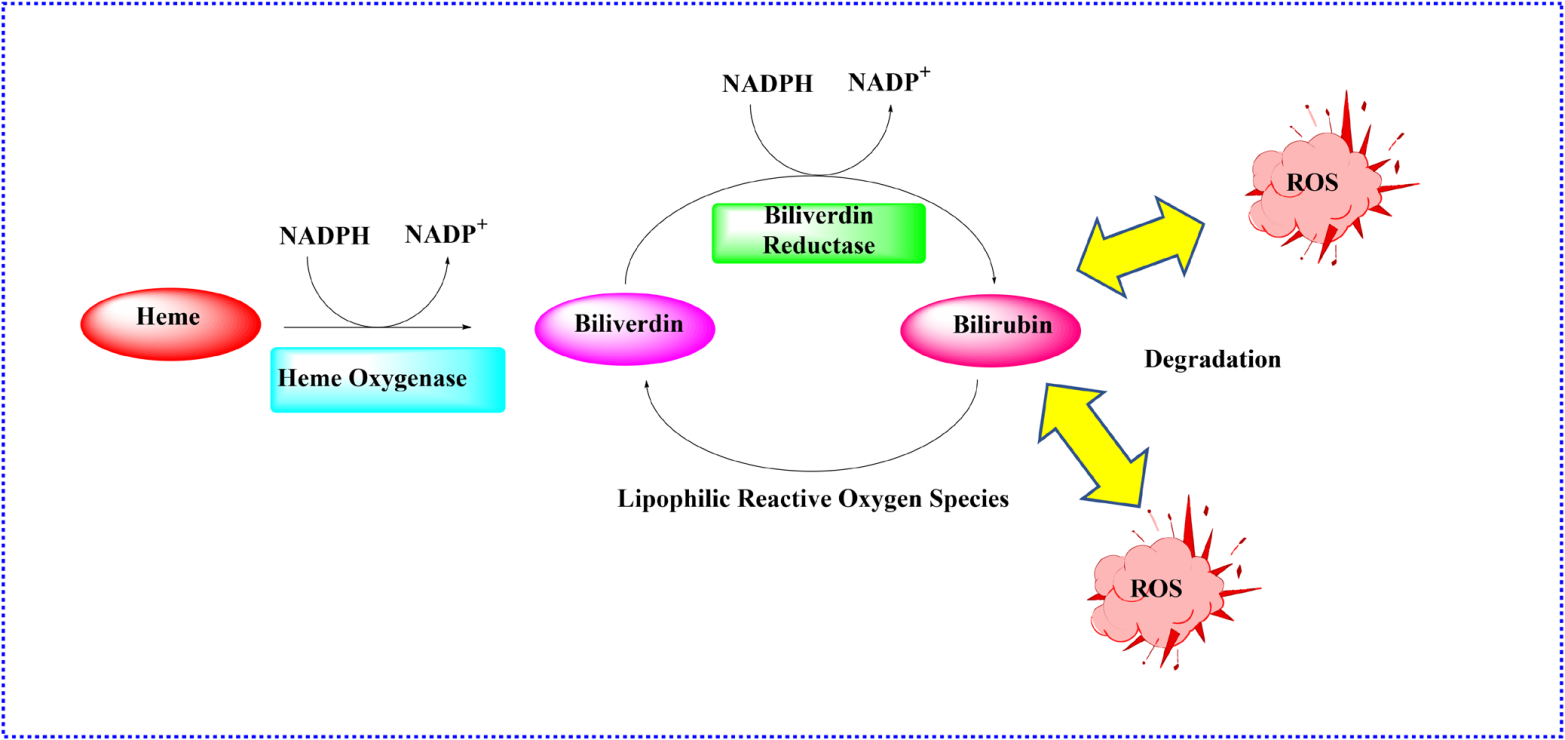
Schematic representation of the heme oxygenase (HMOX) pathway and bilirubin synthesis. HMOX catalyzes the breakdown of heme into biliverdin, carbon monoxide (CO), and ferrous iron (Fe^2+^). Biliverdin is subsequently reduced to bilirubin by biliverdin reductase (BVR). Bilirubin acts as a powerful antioxidant by neutralizing reactive oxygen species (ROS), and it can be recycled back to biliverdin, establishing a redox cycle. CO also exerts cytoprotective effects by modulating inflammation and apoptosis. This pathway plays a critical role in maintaining redox homeostasis, particularly under oxidative stress conditions such as intense physical exercise.

Bilirubin, derived from heme degradation, surpasses vitamins E and C in antioxidative potency. The heme oxygenase system (HMOX–CO–bilirubin) exerts anti‐inflammatory and cytoprotective effects, particularly in cardiovascular and immune contexts. Bilirubin cycles with biliverdin through biliverdin reductase, amplifying its antioxidant capacity (Figure 8) (Nitti et al. 2020; Zhang et al. 2022; Kumari et al. 2023).

Vitamin C is a water‐soluble antioxidant that donates electrons to neutralize ROS and regenerate vitamin E. Vitamin E, lipid‐soluble, protects membranes from peroxidation and regulates lipid signaling. Their combined activity supports membrane and cellular stability (Kaźmierczak‐Barańska et al. 2020; Shastak et al. 2023).

Uric acid (UA) acts as an extracellular antioxidant but can exert pro‐oxidant effects intracellularly by activating NADPH oxidase and inhibiting nitric oxide availability, contributing to oxidative damage in certain contexts (Ye et al. 2023).

Alpha‐lipoic acid (ALA), a mitochondrial cofactor, exhibits potent antioxidant and anti‐inflammatory properties. It has therapeutic relevance in conditions such as diabetes, neurodegeneration, and cardiovascular aging (Gherghina et al. 2022).

## 4 Role of Oxidative Stress in Exercise

Exercise is one of the most common physiological situations causing oxidative stress. It can be observed that exercise causes an increase in oxidative stress and a decrease. Oxidative stress ensues following intense training with productions of active oxygen/nitrogen species that exceed the organism's ability to remove them. Although some level of ROS generation may be necessary for the efficient production of force‐generating capacity within muscles, it seems that excessive ROS levels tend to lead to contractile dysfunction (Furrer et al. 2023). Jackson (2024) has proposed that exercise‐induced mitochondrial biogenesis does not require ROS, but low levels of ROS derived from regular physiological movement/contraction can be a signaling mechanism to stimulate redox‐sensitive signaling pathways and control the adaptive process. Evidence suggests that exercise intensity and duration are related to oxidative stress in humans, which has been supported by numerous studies (Thirupathi, Pinho, et al. 2021; Thirupathi, Wang, et al. 2021). These are of severe nature compared to the moderate regular aerobic exercise ones; however, increased post‐exercise oxidative stress was reported in pathophysiological studies with intense or untrained people. Moreover, regular chronic exercise may enhance certain antioxidant defense systems to a level that might offset mitochondrial oxidative damage (Mason et al. 2020).

Numerous studies indicate that elevated oxygen consumption during exercise correlates with an increase in ROS generation. Oxidative stress arises when the antioxidant system's capacity is inadequate to neutralize ROS generated during exercise. In the last four decades, numerous studies have examined the impact of exercise‐induced oxidative stress. Research has demonstrated that consistent physical activity can enhance the body's antioxidant system and bolster its resilience to oxidative stress (Radak et al. 2008). Regular engagement in physical activity is beneficial for overall health, reducing the risk of cancer, cardiovascular conditions, diabetes, and other chronic diseases (Friedenreich et al. 2021). Elevated biomarkers reflecting oxidative damage in blood and skeletal muscle provide indirect evidence of oxidative stress triggered by exercise. During physical exercise, the contraction of skeletal muscles generates free radicals, while increased oxygen consumption leads to a substantial production of ROS. A lack of adequate antioxidant defenses in the body contributes to oxidative damage to cells and tissues (Lu et al. 2021).

Physical exercise is a complex biological process that continuously challenges the body's oxidation‐antioxidant balance across cells, tissues, and organs while maintaining biological homeostasis. The influence of exercise on oxidative stress can manifest as either an acute or chronic response (Hawley et al. 2014). Acute responses reflect insufficient adaptation, which may lead to oxidative damage if not managed properly. Consequently, ensuring adequate rest following exercise is essential for restoring equilibrium. The cycle of balance disruption and subsequent restoration strengthens the body's ability to cope with oxidative stress. Consistent physical activity plays a pivotal role in augmenting the body's innate antioxidant defense mechanisms (Nieman and Wentz 2019).

Regular moderate‐intensity exercise has been shown to boost the activity of endogenous antioxidant enzymes including CAT, GPx, and SOD. By improving the body's resilience to prolonged low‐to‐moderate exposure to ROS, exercise facilitates fundamental adaptations related to redox processes, including the activation of repair mechanisms that mitigate oxidative damage. This adaptation also involves an increase in myocellular antioxidant capacity, contributing to a reduction in ROS levels (Verhaegen et al. 2022). Furthermore, enhancing ROS production in active skeletal muscle through the modulation of muscular contraction is crucial for exercise adaptation. Endurance running is deemed crucial for survival in human development as it can elicit exercise‐induced contractile responses via metabolic and redox challenges (Bouviere et al. 2021). Nevertheless, contemporary lifestyles have led to diminished physical activity, hence impeding human adaptability in redox metabolism and homeostasis. Fundamental research indicates that a minimum of 30 min of moderate‐intensity exercise daily is crucial for sustaining good health and mitigating potential illness risks (Qiu et al. 2023).

Zarrindast et al. (2021) reported that 8 weeks of moderate‐intensity aerobic training, conducted both on land and in water, can decrease oxidative stress and enhance antioxidant status. Additionally, Done and Traustadóttir (2016) observed an increase in antioxidant gene transcripts, demonstrating that regular aerobic exercise enhances the body's tolerance to oxidative stress. However, Estébanez et al. (2022) found that aerobic exercise does not produce significant effects on oxidative stress markers in the elderly population. Leelarungrayub et al. (2011) found that choreography dance at moderate intensity for 6 weeks may decrease MDA levels and increase total antioxidant (TAC) increasing properties in sedentary women. In principle, moderate to transient generation of ROS during a short‐term aerobic regimen could activate proadaptive signaling cascades and protect against subsequent beating‐related stressors. On the other hand, continuous moderate values of ROS production lasting several hours of production during short duration, high‐intensity training may lead to tissue and structural damage.

Regular and prolonged resistance training has been shown to boost the activity of antioxidant enzymes. In this regard, Vezzoli et al. (2019) found that 12 weeks of moderate‐intensity resistance training can lower the production of ROS and oxidative stress. It has been suggested that moderate‐intensity resistance training can counteract anabolic resistance and enhance protein synthesis in older adults. Furthermore, da Silva et al. (2016) reported that 6 months of resistance training can enhance the body's response to oxidative stress, potentially improving both performance and overall health. Their study revealed an increase in CAT activity without any significant changes in SOD activity. Additionally, Motameni et al. (2020) showed that three types of acute resistance exercise (hypertrophy, strength, and power) do not increase oxidative stress in women who regularly participate in resistance training. No substantial alteration in H_2_O_2_ and MDA levels was seen as a result of the resistance exercise. Evidence indicates that variations in training intensity and volume whether—characterized by high volume and low intensity or low volume and high intensity are—likely to positively influence the increase in GSH concentration (Çakir‐Atabek et al. 2010).

Beyond their role in ATP production during aerobic metabolism, mitochondria are recognized as the primary intracellular source of pro‐oxidants. There are multiple redox centers in the mitochondrial electron transport chain that make electron leakage to oxygen and reduce it to O_2_^−^. This is related to the branching of oxidative chain processes, which is a forerunner of subsequent ROS. Aerobic exercise is known to induce levels of the pro‐oxidant that are much higher than physical activity of the anaerobic type; in fact, exercise‐induced oxidative stress appears to depend on several factors, such as the type of activity, duration, and intensity (Gomes et al. 2012). Suzuki et al. (2003) claimed that increasing the intensity of exercise boosts the body's internal antioxidant defenses, while NADPH transplantation directly spares plasma antioxidants, suggesting that these results reflect elevated ROS production that induces antioxidant release and subsequent modulation of ROS during high‐intensity exercise. Redox state‐related health effects occur after high‐intensity exercise in some instances. Azizbeigi et al. (2014) reported that the resistance, endurance, and concurrent training (resistance + endurance) reduced MDA and improved enzymatic antioxidant capacity (SOD, and GPx) in untrained males. Only [Endurance] and [Concurrent] groups had significantly raised levels of TAC. They emphasized that whether the increase in enzyme activity in the concurrent group was primarily due to adaptations to endurance, to resistance exercise, or to both was unclear, and what would have had the greatest influence.

Identifying disturbances in the redox equilibrium caused by phenolic compounds is essential, particularly for athletes. It facilitates information regarding the state of the organism post‐training, adaptation to stress circumstances, and the necessity for antioxidant supplements (Cuyul‐Vásquez et al. 2020). Evidence indicates that a moderate increase in ROS/RNS can facilitate adaptation to exercise‐induced oxidative stress through efficient signal transduction. Conversely, elevated levels of oxidative stress may lead to contractile muscle dysfunction, heightened fatigue, prolonged recovery periods, and diminished athletic performance (Carneiro et al. 2021). Research suggests that high‐load resistance exercise can promote mitochondrial biogenesis and improve respiratory performance through the synthesis of myofibrillar or mitochondrial proteins. These effects depend on an individual's training status and the intensity of the exercise performed (Groennebaek et al. 2018). Low‐load resistance exercise promotes muscle growth and mitochondrial adaptations in skeletal muscle without significantly increasing myofibrillar content. The resulting increase in mitochondrial quantity helps maintain redox balance more effectively in trained individuals compared to untrained individuals when performing exercises of similar intensity (Cobley et al. 2014).

Exercise performance training seeks to enhance muscular strength, speed, agility, and injury prevention. Antioxidants and exercise training are essential for enhancing endurance capacity and athletic performance. To reduce oxidative stress, enhance exercise performance, and avert muscle damage caused by ROS, endurance athletes frequently utilize exogenous antioxidant supplements (Clifford et al. 2020). This method is based on athletes' expectation that supplement consumption may postpone muscular tiredness, facilitate recovery, and boost athletic performance. Consequently, antioxidant supplementation has garnered interest from athletes, researchers, and the supplement industry. A key question is whether the use of antioxidant supplements, such as phenolic compounds, can improve exercise performance outcomes and what effects (positive or adverse) long‐term supplementation may have on skeletal muscle adaptation to endurance or resistance training (Somerville et al. 2017). Research indicates that antioxidant supplementation in physically active individuals boosts vascular function, preserves redox homeostasis, mitigates muscular fatigue, and improves endurance performance. Conversely, increasing evidence indicates that high dosages of antioxidants adversely affect skeletal muscle responses to endurance training (Gomez‐Cabrera et al. 2008).

## 5 Polyphenolic Compounds

**FIGURE 9:**
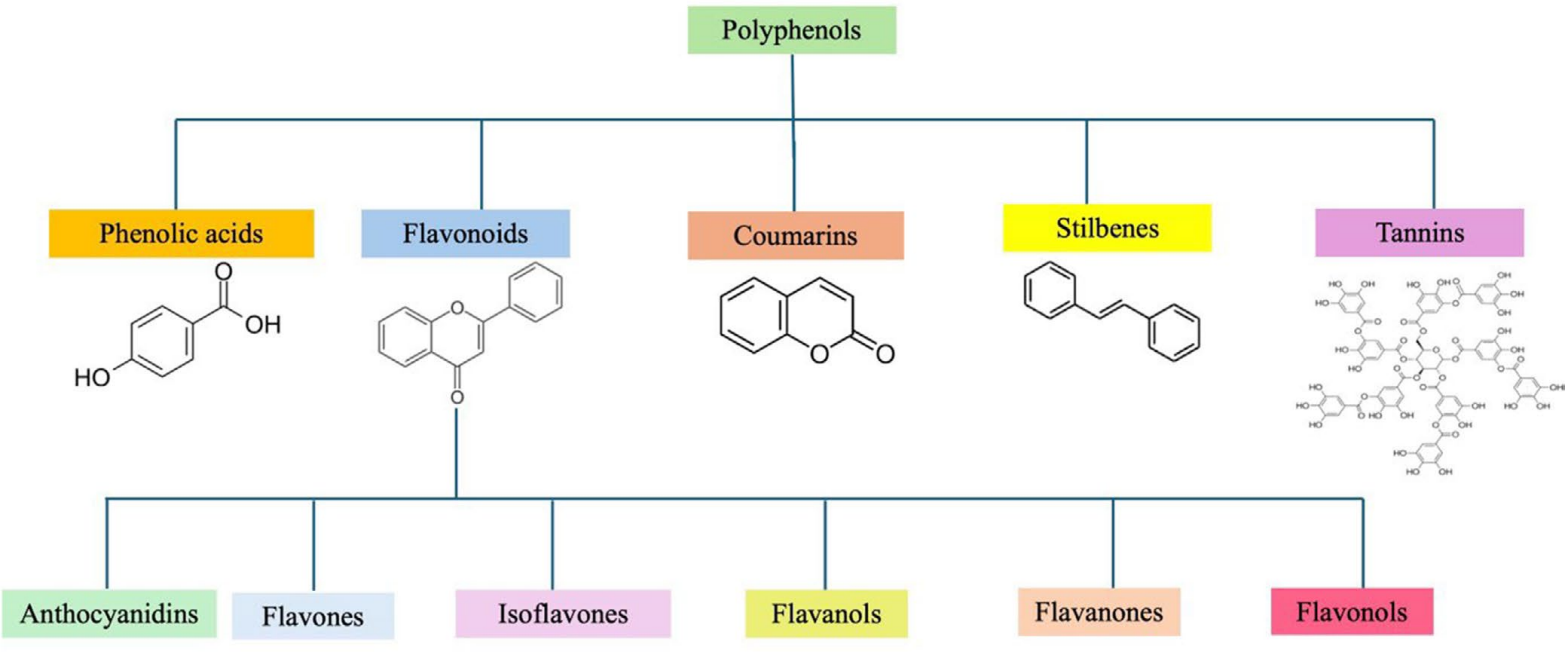
Categorization of phenolic compounds.

Polyphenolic compounds (PCs) are a broad and numerous class of natural antioxidants found in a variety of fruits, plant‐derived beverages (such as tea and juice), vegetables, seeds, and other plant‐based meals (Hussain et al. 2021; Rudrapal et al. 2022; Egbuna et al. 2023). In plants, these molecules play a crucial role in several functions, including pigmentation, development, reproduction, and defense against diseases or predators (Aslan and Beydemir 2017; Topal and Gulcin 2022). Evidence has identified a list of 100 dietary products that are highest in PCs, with concentrations varying from 15,000 mg/100 g in cloves to 10 mg/100 mL in red wine, and as many as 89 foods and beverages containing over 1 mg of total PCs per serving (Pérez‐Jiménez et al. 2010). PCs include one or more hydroxyl groups (–HO) bonded to a benzene ring (Rudrapal et al. 2024). Five primary classes of phenolics are identified based on chemical structure among 10 or more types: phenolic acids (e.g., caffeic acid, gallic acid), flavonoids, stilbenes (e.g., resveratrol), lignins (cross‐linked phenolic polymers), and condensed tannins (large molecules derived from phenolic acids) (Abotaleb et al. 2020) (Figure 9).

Due to their structural diversity and biological functions, phenolic compounds have garnered significant attention in fields such as pharmacology, food science, and medicine. Their potent antioxidant properties, which enable them to reduce oxidative stress and neutralize free radicals, make them essential contributors to human health (Karagecili et al. 2023; Tel et al. 2025). The antioxidant potential of phenolic compounds is primarily attributed to their ability to donate, chelate metal ions, hydrogen electrons or atoms, and upregulate endogenous antioxidant defenses (Aslan et al. 2024). These properties position phenolics as protective agents against various oxidative stress‐related diseases, including cancer, cardiovascular disorders, neurodegenerative diseases, and diabetes (Aslan et al. 2025). The antioxidant activity of phenolic compounds is intricately linked to their chemical structure. The ‐OH group is a key functional unit, capable of donating a hydrogen atom to neutralize free radicals. Additionally, the delocalized π‐electrons of the aromatic ring stabilize the resulting phenoxyl radical, preventing propagation of radical chain reactions (Guven et al. 2023).

The range of biological activities of PCs is extensive. The specific processes (indirect effects) underlying the biological effects of PCs are associated with interactions with proteins and are contingent upon the functions of these proteins and the metabolites resulting from their biotransformation (Demirtas et al. 2025). This pathway encompasses interactions with enzymes implicated in inflammation, including phospholipases A2 (PLA2), COX1/2, and LOX, subsequently leading to a reduction in O_2_^·−^ levels and NO· availability, as well as the regulation of blood pressure. Another mechanism involves the modulation of redox‐sensitive transcription factors, such as nuclear factor kappa B (NF‐κB), which is implicated in intracellular signaling cascades, and activator protein 1 (AP‐1), or in interaction with estrogen receptors, functioning as either estrogen agonists or antagonists due to the structural similarity of isoflavones to estrogens (Bowtell and Kelly 2019). Certain investigations indicated that PCs at physiological levels may inhibit indicators of ROS, which are secondary products of the inflammatory response to external stimuli (Yahfoufi et al. 2018).

## 6 Polyphenolic Compounds Supplementation and Exercise

### 6.1 Curcumin Supplementation

Curcumin (CUR), the primary bioactive polyphenol in turmeric ( *Curcuma longa* ), has garnered considerable attention for its antioxidant, anti‐inflammatory, and exercise recovery‐enhancing properties. Its chemical structure, featuring two methoxy phenolic groups and a β‐diketone moiety, allows curcumin to effectively scavenge ROS such as O_2_^·−^, OH, and H_2_O_2_, while also modulating redox‐sensitive signaling pathways (Hewlings and Kalman 2017). Mechanistically, curcumin exerts its antioxidant effect predominantly through the activation of the Nrf2 pathway, resulting in the transcriptional upregulation of endogenous antioxidants such as HO‐1, CAT, and SOD (Patel et al. 2020). Moreover, it inhibits the NF‐κB pathway, which plays a critical role in inflammation, especially during strenuous or prolonged exercise (Qiu et al. 2020).

In human trials involving athletes and physically active individuals, CUR supplementation has been shown to reduce delayed onset muscle soreness (DOMS), oxidative stress markers, and inflammatory cytokines. For example, Drobnic et al. (2014) administered 150 mg/day of a bioavailable curcumin formulation (Meriva) for 8 days and observed reduced muscle soreness and improved recovery in healthy male volunteers after eccentric exercise. Other studies have used doses ranging from 200 mg to 1000 mg/day, particularly in studies on inflammation, mitochondrial function, and exercise‐induced fatigue (Hewlings and Kalman 2017).

CUR is generally considered safe in humans, with daily doses up to 2000 mg/day well tolerated in clinical studies (Cheng et al. 2001). Researchers examined the potential of CUR, a polyphenolic compound with strong anti‐inflammatory properties, to mitigate muscle damage following eccentric exercise. Fourteen young, untrained men participated in two exercise sessions involving 50 maximal eccentric contractions of the elbow flexors, with each session targeting one arm. The participants were administered 150 mg of CUR or a PLA in a randomized, crossover design. Key variables such as maximal voluntary contraction (MVC) torque, muscle soreness, serum creatine kinase (CK) activity, and inflammatory markers were assessed before and after exercise. The findings indicated that CUR reduced the decline in MVC torque and peak CK levels compared to the PLA, suggesting its efficacy in attenuating certain markers of muscle damage. However, no significant changes were observed in inflammatory markers such as IL‐6 and TNF‐α (Tanabe et al. 2015).

Sayevand et al. (2022) examined the effects of 10 weeks of moderate‐intensity aerobic exercise and CUR supplementation, both individually and in combination, on ischemia–reperfusion‐induced myocardial injury in rats. The researchers found that both treatments, when applied separately, reduced infarct size and decreased mRNA expression of amyloid precursor protein and enzymes involved in amyloid processing (β‐secretase‐1, presenilin‐1 and ‐2).

Akazawa et al. (2012) explored the impact of CUR ingestion and aerobic exercise on vascular endothelial function in postmenopausal women, as assessed by flow‐mediated dilation. Thirty‐two participants were divided into three groups: control, exercise, and CUR. Over an 8‐week period, one group consumed CUR supplements while another group participated in moderate aerobic exercise training. Flow‐mediated dilation (FMD) was assessed both before and after the interventions. Both the CUR and exercise groups exhibited significant and comparable improvements in FMD, whereas no changes were detected in the control group.

Delecroix et al. (2017) examined the impact of CUR and piperine supplementation on recovery following exercise‐induced muscle injury in professional rugby athletes. Employing a randomized crossover design, 10 athletes ingested curcumin and piperine or a PLA 48 h before and following muscle‐damaging activity. Assessments of knee extensor peak torque, sprint performance, jump performance, CK concentrations, and muscle soreness were conducted immediately post‐exercise and at 24, 48, and 72 h thereafter. The results indicated that exercise markedly diminished muscle function and elevated CK levels, signifying muscle injury. Significantly, 24 h after exercise, sprint power production was somewhat superior in the CUR plus piperine group relative to the PLA group. Nonetheless, no more notable impacts were seen between the two circumstances.

Huang et al. (2015) assessed the impact of CUR administration on exercise performance and tiredness in male ICR mice. During a 4‐week period, mice were categorized into four groups, each receiving different dosages of CUR, and were subjected to physical trials to assess forelimb grip strength, exhausting swimming duration, and fatigue‐related biomarkers. The results indicated that CUR supplementation enhanced grip strength and endurance in a dose‐dependent fashion. Furthermore, it reduced biomarkers linked to physical tiredness, including serum lactate, ammonia, blood urea nitrogen (BUN), and indicators of tissue damage such as AST, ALT, and CK. CUR also increased muscle glycogen levels, a crucial energy reservoir for physical activity, with few negative repercussions.

### 6.2 Quercetin Supplementation

Quercetin (QUR) is a widely distributed flavonol present in various plant‐based foods such as onions, apples, red grapes, and green tea. It exhibits potent antioxidant properties by both direct radical scavenging and activation of redox‐sensitive signaling pathways (Ekinci Akdemir, Gülçin, Karagöz, and Soslu 2016; Ekinci Akdemir, Gülçin, Karagöz, Soslu, and Alwasel 2016). Mechanistically, QUR stimulates the Nrf2/ARE pathway, which enhances the expression of endogenous antioxidant enzymes including HO‐1, SOD, and GPx (Costa et al. 2016). It also directly neutralizes O_2_^·−^ and reduces inducible nitric oxide synthase (iNOS) activity, thereby decreasing NO and ONOO^−^ levels (Li, Jiang, et al. 2023; Li, Wang, et al. 2023). In exercise physiology, quercetin has been shown to improve endurance capacity, attenuate muscle damage, and promote mitochondrial biogenesis by upregulating Sirt1 signaling pathways (Casuso et al. 2014). These effects are particularly relevant under conditions of high intensity or prolonged exercise where oxidative damage is elevated. However, studies also suggest that excessive intake of QUR (above 1000 mg/day) may exert pro‐oxidant effects by generating semiquinone radicals and interfering with adaptive oxidative stress signaling (Boots et al. 2008). Clinical studies typically use doses ranging from 500 to 1000 mg/day in healthy adults. For elderly populations with reduced endogenous antioxidant capacity, slightly higher doses (up to 1200 mg/day) may be considered under medical supervision (Heinz et al. 2010). Taken together, the evidence highlights the context‐dependent efficacy of QUR, particularly in populations with varying baseline antioxidant capacities and physical activity habits.

Davis et al. (2010) demonstrated the beneficial impact of QUR on the VO_2max_ and endurance in individuals who did not engage in regular physical activity. The baseline VO_2max_ and the riding duration necessary for exhaustion were evaluated to measure endurance. Over a period of 7 days, one cohort of subjects received a supplementation of 500 mg of QUR bi‐daily, while the other cohort was given a PLA. The research was executed as a randomized, double‐blind trial. Individuals administered polyphenol exhibited a 3.9% enhancement in VO_2max_ relative to the PLA cohort, along with a 13.2% prolongation in the duration before fatigue onset.

Wu et al. (2012) investigated the lipolytic effects of QUR supplementation in swimming mice. The mice were fed a 0.1% flavonoid‐enriched diet for 14 days before undergoing a 60‐min swimming session. A reduction in lactate levels indicated a decrease in glycolysis, potentially as a compensatory response to energy deficiency. This was accompanied by an increase in unsaturated fatty acids, suggesting an enhancement in lipolysis. Gao et al. (2014) evaluated the protective effects of QUR, a flavonoid with antioxidant characteristics, on mitochondrial oxidative stress and dysfunction induced by repeated vigorous exercise. Male BALB/C mice received QUR (100 mg/kg body weight) for 4 weeks and underwent a treadmill training regimen. Intense activity resulted in elevated CK‐MB levels, disordered myofibrils, and enlarged mitochondria. QUR pretreatment, however, markedly mitigated these effects by reducing mitochondrial oxidative stress, preserving GSH levels, and enhancing mitochondrial function. The results indicate that QUR safeguards the myocardium against exercise‐related injury, positioning it as a viable natural approach to mitigate over‐training damage.

Duranti et al. (2018) examined the impact of QUR supplementation on redox homeostasis in healthy subjects during intense eccentric exercise. In a two‐week controlled, randomized crossover experiment, 14 volunteers consumed 1 g/day of quercetin or a PLA. Blood samples were collected before and subsequent to supplementation and after eccentric exercise. The research indicated that QUR markedly decreased lipid peroxidation in erythrocytes and lowered vulnerability to hemolysis induced by oxidative stress. Despite the absence of substantial changes in antioxidant enzyme activity and glutathione homeostasis, QUR enhanced redox equilibrium after exercise by elevating the reduced/oxidized GSH ratio and decreasing TBARs levels in both erythrocytes and plasma. The results indicate that continuous QUR administration may improve the body's capacity to regulate oxidative stress, particularly during intense exercise.

### 6.3 Resveretrol Supplementation

Resveratrol (RES) is a naturally occurring phenolic molecule included in several foods, including red wine, blueberries, peanuts, and grapes. It has several bioactivities, including immunomodulatory, anti‐inflammatory, hypolipidemic, hypotensive, and antioxidant effects, along with efficacy in the prevention and management of obesity, cancer, neurological disorders, and cardiovascular diseases (Meng et al. 2020). While resveratrol is generally considered safe at doses up to 1 g per day, high concentrations may exert pro‐oxidant effects under certain conditions, potentially impairing ROS‐mediated adaptive processes (Edwards et al. 2011). The recommended daily intake ranges between 150–500 mg for healthy adults and up to 750 mg for elderly individuals. It is available in supplemental forms, commonly in 250–1000 mg capsules (Detampel et al. 2012). Beyond its well‐characterized pharmacokinetics and recommended intake, recent evidence has begun to elucidate resveratrol's role in enhancing exercise‐related mitochondrial function and reducing fatigue.

Lou et al. (2023) highlighted the beneficial effects of RES supplementation on mitigating exercise‐induced fatigue and enhancing mitochondrial energy metabolism. Using a rat model, resveratrol was shown to significantly lower plasma blood urea nitrogen, CK activity, and MDA levels in skeletal muscle, indicating a reduction in muscle damage and oxidative stress. Additionally, resveratrol enhanced total SOD activity, suggesting improved antioxidant defense mechanisms. At the molecular level, the upregulation of the SIRT1‐PGC‐1α‐ and NRF1 signaling pathway suggests a potential mechanism by which resveratrol enhances mitochondrial biogenesis and function.

Xu et al. (2023) investigated the protective effects of RES against high‐intensity exercise‐induced intestinal damage in mice, revealing its potential to mitigate inflammation, oxidative stress, intestinal barrier disruption, and ferroptosis. High‐intensity exercise caused significant intestinal damage, marked by elevated levels of inflammatory cytokines (TNF‐α, IFN‐γ, IL‐6) and reduced anti‐inflammatory IL‐10. Oxidative stress indicators were similarly affected, with increased H_2_O_2_ and MDA levels alongside decreased CAT and GSH activities. Additionally, exercise compromised intestinal barrier integrity, as evidenced by increased gut permeability, higher intestinal fatty‐acid binding protein concentrations, and reduced expression of tight junction proteins. RES treatment (15 mg/kg/day) effectively alleviated these adverse effects. It reduced inflammation and restored antioxidant activity by enhancing CAT and GSH.

### 6.4 Myricetin Supplementation

Myricetin (MYR), commonly present in red wine, vegetables, and berries, has several biological roles. Research indicates that myricetin might mitigate detrimental oxidative stress by modulating the antioxidant enzyme system, with implications for cardiovascular consequences (Hassan et al. 2017). MYR enhances the cellular antioxidant response by activating the Nrf2 pathway, thereby upregulating downstream enzymes, such as HO‐1, CAT, and GPx. In addition to its direct scavenging effects on O_2_^·−^ and H_2_O_2_, MYR modulates inflammation by inhibiting NF‐κB signaling and suppressing inducible iNOS expression, thus reducing NO and ONOO^−^ generation (Zhang et al. 2019). In exercise‐related contexts, MYR supplementation has been shown to preserve mitochondrial function and improve physical endurance.

Recent research has explored strategies to counteract hypoxia‐induced exercise intolerance, with a focus on MYR, a flavonoid found in fruits and vegetables. This study evaluated the effects of myricetin supplementation on acute hypoxia‐induced exercise intolerance both in vivo and in vitro. Using a rat model subjected to hypobaric hypoxia (simulating an altitude of 5000 m), it was demonstrated that myricetin significantly improved exercise performance, as assessed through run‐to‐fatigue testing. Mechanistically, MYR was shown to mitigate hypoxia‐induced mitochondrial dysfunction in skeletal muscle cells. It preserved the activity of respiratory chain complexes, membrane potential, mtDNA content, and mitochondrial structure. These effects were attributed to myricetin's ability to enhance mitochondrial biogenesis through the upregulation of key biogenesis regulators. Importantly, the activation of AMP‐activated protein kinase (AMPK) emerged as a critical pathway mediating these protective effects (Zou et al. 2015). Similarly, Jung et al. (2017) found that myricetin treatment (50 mg/kg/day) improved aerobic endurance in mice by activating SIRT1 and PGC‐1α, resulting in increased mitochondrial biogenesis and oxygen consumption rate.

Li, Jiang, et al. (2023) and Li, Wang, et al. (2023) employed a mouse model to evaluate the effects of MYR following high‐intensity exercise (HIE) and examined its underlying mechanisms. The findings revealed that myricetin significantly alleviated myocardial ultrastructural damage, reduced markers of myocardial injury, improved cardiac function, and decreased ischemic/hypoxic areas in the myocardium. Notably, MYR enhanced the expression of connexin 43 (CX43), a critical protein for cardiac electrical and metabolic coupling. Mechanistic analysis, integrating metabolomics and network pharmacology, identified potential therapeutic targets of MYR, including the downregulation of MAOB and PTGS2 and the upregulation of EGFR and MAP2K1.

### 6.5 Hesperidin Supplementation

Hesperidin (HES) is a flavanone classified within the diverse and widely prevalent group of plant phenolics known as flavonoids, found in high concentrations in citrus fruits. Natural sources of hesperidin include citrus fruits such as oranges and lemons, with juice concentrations reaching up to ~500 mg/L (Ekinci Akdemir, Gülçin, Karagöz, and Soslu 2016; Ekinci Akdemir, Gülçin, Karagöz, Soslu, and Alwasel 2016). Recommended intake levels range from 300–600 mg/day for healthy adults and up to 1000 mg/day for individuals with increased oxidative stress, such as athletes or elderly populations (EFSA Panel on Nutrition 2024).

Evidence suggests that HES supplementation exhibits insulin‐sensitizing, anti‐inflammatory, lipid‐lowering, and neuroprotective effects (Imperatrice et al. 2022). It exhibits strong antioxidant and anti‐inflammatory properties by activating the Nrf2/ARE signaling pathway (Mahmoud et al. 2017). In vivo studies have demonstrated that HES effectively scavenges H_2_O_2_, restores mitochondrial membrane potential, and reduces malondialdehyde (MDA) levels, suggesting a protective role against oxidative cell damage (Iskender et al. 2017).

HES also enhances NO bioavailability by modulating endothelial nitric oxide synthase (eNOS) activity, improving vasodilation and peripheral blood flow during exercise (Imperatrice et al. 2022), while simultaneously reducing ROS production and inflammation (Buzdağlı et al. 2023).

A single dose of HES supplementation (500 mg) was found to enhance the activity of the endogenous antioxidant enzyme CAT in venous blood samples collected following intense physical exertion, as assessed by a Wingate test on a cycle ergometer in male amateur cyclists. However, other endogenous antioxidant markers, including SOD and GSH, as well as lipid oxidation markers such as TBARS, did not exhibit significant changes between the HES and control groups. Notably, a declining trend in SOD levels was observed within the intervention group, though it did not reach statistical significance (Martínez‐Noguera et al. 2019). Similarly, 6 weeks of hesperetin supplementation (50 mg·kg^−1^·day^−1^), the primary metabolite of HES, significantly increased the GSH/GSSG ratio and enhanced running performance (exercise duration) in aged mice (Biesemann et al. 2018).

In a study involving healthy soccer players, acute supplementation with 217 mg of HES resulted in a reduction of the lipid peroxidation marker MDA in plasma following exercise. Additionally, plasma TAS increased significantly post‐exercise in both the HES and PLA groups, although no significant differences were observed between the two groups regarding TAS levels (Boussetta et al. 2020). Another study, Ruiz‐Iglesias et al. (2020) aimed to evaluate the impact of oral HES supplementation on the systemic immune response in rats subjected to intensive training and exhausting exercise. Female Wistar rats were randomized into either an intensive training group or a sedentary control group. Intensive training consisted of treadmill running 5 days per week, including two exhaustion tests, over a five‐week period. During this time, rats were administered 200 mg/kg of HES or vehicle via oral gavage three times per week. At the conclusion of the training regimen, blood, thymus, spleen, and macrophages were collected at three time points: pre‐exercise, immediately post‐exercise, and 24 h after an additional final exhaustion test. HES supplementation was found to enhance natural killer cell cytotoxicity and increase the proportion of phagocytic monocytes.

### 6.6 Gallic Acid Supplementation

Gallic acid (GA) is a naturally occurring compound found in a variety of sources, including beverages, nuts, pineapples, cucumber, red grapes, bananas, bark, tea leaves, oak strawberries, apple skins, lemons, and numerous medicinal herbs. Furthermore, GA has been shown to induce apoptosis in cancer cells and GPx levels, underscoring its role as an effective antioxidant (Gangadharan et al. 2024). The recommended intake of GA for humans is estimated to range from 100 to 300 mg/day in supplementation studies, with doses up to 500 mg/day being considered safe and effective in conditions of elevated oxidative stress (Yu et al. 2025). It exhibits potent antioxidant properties by acting as a hydrogen donor and metal ion chelator, thus neutralizing free radicals (Hadidi et al. 2024). Moreover, GA activates the Nrf2/Keap1 pathway, leading to upregulated expression of endogenous antioxidant enzymes (Badawy et al. 2024).

In cellular and animal models, GA supplementation has been shown to mitigate exercise‐induced oxidative stress by reducing lipid peroxidation, restoring mitochondrial membrane potential, and preventing DNA fragmentation in muscle tissues. Furthermore, GA possesses anti‐inflammatory properties by inhibiting the NF‐κB pathway, reducing the expression of COX‐2, TNF‐α, and IL‐6, which are upregulated during intense physical stress (Chu et al. 2024). This makes it a promising compound for enhancing recovery and reducing tissue damage in athletes undergoing high‐volume training.

A recent experimental study investigated the effects of physical exercise and GA on passive avoidance memory in rats. Forty‐six rats were randomized into six groups: a control group, groups treated with 10 or 20 mg/kg GA, a group subjected to treadmill exercise alone, and groups combining treadmill exercise with 10 or 20 mg/kg GA. The interventions were administered over 10 days. Passive avoidance memory was assessed using a shuttle box, while serum and brain antioxidant capacity and MDA levels were measured. The findings revealed a significant increase in secondary latency in the shuttle box for all treatment groups, with the most pronounced effects observed in the combined exercise and GA groups. Rats treated with GA alone (10 or 20 mg/kg) also demonstrated improved memory performance (Salehi et al. 2019). Another study evaluated the effects of oral GA supplementation, both alone and in combination with exercise, on nerve conduction velocity (NCV) and sensory and motor functions in a rat model of sciatic nerve crush injury. Seventy adult male Wistar rats were divided into seven groups, including controls, vehicle‐treated crush injury, three GA‐treated groups (50, 100, and 200 mg/kg), exercise alone, and exercise combined with the highest GA dose (200 mg/kg). The interventions lasted for 21 days, and outcomes such as pain reflex, sciatic nerve conduction velocity (SNCV) and motor coordination were assessed. The results showed that sciatic nerve crush significantly increased pain thresholds and reduced motor function and SNCV compared to controls. While pain reflex latency remained unchanged with treatment, motor coordination and SNCV improved significantly in the groups treated with 200 mg/kg GA and the combination of exercise and GA (*p* < 0.05, *p* < 0.01 vs. vehicle‐treated crush group) (Hajimoradi et al. 2015).

### 6.7 Caffeic Acid Phenethyl Ester Supplementation

Caffeic acid phenethyl ester (CAPE), a bioactive component derived from propolis, has gained significant attention due to its antioxidant, antiproliferative, anti‐inflammatory, antiviral, and antifungal properties (Göçer and Gülçin 2011). CAPE has been shown to suppress the expression of inducible iNOS and COX‐2, thereby reducing the production of NO and prostaglandin E2, both of which are central mediators of inflammation (Celik and Erdogan 2008). Additionally, CAPE downregulates pro‐inflammatory cytokines such as TNF‐α, IL‐1β, and IL‐6, mainly by inhibiting the NF‐κB signaling pathway (Jang et al. 2023). Beyond its anti‐inflammatory effects, CAPE also inhibits the activity of NADPH oxidase isoforms NOX‐2 and NOX‐4, leading to a reduction in the generation of ROS and attenuation of oxidative stress (Zhang et al. 2024). Collectively, these actions suggest that CAPE may confer protective benefits in exercise‐induced inflammation and muscle damage by modulating both pro‐inflammatory mediators and oxidative pathways.

Coban et al. (2017) investigated the effects of CAPE on exercise‐induced oxidative stress. Using Sprague–Dawley rats (250–300 g), the experiment divided the animals into four groups and examined biochemical markers and DNA damage using a comet assay. Acute swimming exercise resulted in increased oxidative stress, as evidenced by DNA damage in mononuclear leukocytes and elevated levels of 8‐OHdG. CAPE supplementation significantly reduced MDA and 8‐OHdG levels (*p* < 0.05), suggesting its protective role against exercise‐induced oxidative stress. Another study, Akil et al. (2016) investigated the effects of CAPE supplementation on liver element levels in rats subjected to exercise. Thirty‐two male Sprague–Dawley rats were divided into four groups: (1) CAPE‐only group receiving 10 μmol/kg/day intraperitoneally (IP) for 4 weeks without exercise, (2) CAPE plus exercise group receiving the same CAPE dose with swimming exercise at the end of the 4th week, (3) general control group, and (4) swimming control group. CAPE was dissolved in 10% ethyl alcohol for administration. At the conclusion of the experiment, liver tissue samples were analyzed for magnesium (Mg), cadmium (Cd), chromium (Cr), zinc (Zn), copper (Cu), calcium (Ca), iron (Fe), potassium (K), and sodium (Na) levels. Results indicated that CAPE supplementation and exercise induced changes in Cr, Fe, Na, Ca, and Zn levels in liver tissues but did not significantly affect Mg, K, Cu, or Cd levels. These findings suggest that CAPE supplementation may help maintain liver element homeostasis, particularly for elements affected by exercise.

## 7 Conclusion

Exercise induces oxidative stress by increasing the generation of reactive oxygen and nitrogen species (ROS/RNS), which can impair muscle function, delay recovery, and promote cellular damage. However, at physiological levels, ROS also serve as important signaling molecules that mediate beneficial adaptations such as mitochondrial biogenesis, angiogenesis, and antioxidant enzyme upregulation. This duality highlights the complex relationship between oxidative stress and exercise‐induced adaptations. Phenolic compounds, widely found in plant‐based foods, exert significant antioxidant and anti‐inflammatory effects through multiple mechanisms. These include direct scavenging of free radicals, chelation of pro‐oxidant metals, inhibition of redox‐sensitive pro‐inflammatory pathways (e.g., NF‐κB), and activation of cellular defense systems via the Nrf2‐ARE pathway. Specific polyphenols such as curcumin, quercetin, resveratrol, and myricetin have demonstrated promising roles in enhancing muscle recovery, reducing exercise‐induced damage, and improving endurance and metabolic function. However, recent evidence suggests that excessive or chronic supplementation with phenolics may blunt the beneficial stress signaling required for training adaptations, especially when ROS levels are suppressed below physiological thresholds.

In conclusion, phenolic compounds represent valuable modulators of redox balance in exercise physiology, but their application must be evidence‐based, context‐sensitive, and individualized. Future research should focus on long‐term outcomes, optimal dosing regimens, interactions with other antioxidants, and phenotypic responses to supplementation across diverse populations.

## 8 Limitations and Directions for Future Research

While this review presents current evidence on the role of phenolic compounds in modulating exercise‐induced oxidative stress, several limitations should be acknowledged. The heterogeneity of study designs, dosages, and phenolic sources makes it difficult to generalize the findings across populations and exercise modalities. In many cases, the bioavailability and metabolism of phenolic compounds are not thoroughly evaluated, limiting the translational value of results. Future research should focus on dose–response relationships and long‐term effects of individual phenolics on exercise adaptations, particularly at the mitochondrial and epigenetic levels. Human clinical trials with standardized phenolic formulations and controlled dietary interventions are needed to validate current experimental findings. Additionally, the potential pro‐oxidant effects of certain phenolics under high‐dose or chronic conditions should be systematically explored. Personalized approaches, considering genetic polymorphisms and gut microbiota composition, may also help clarify interindividual variability in phenolic efficacy. Overall, integrated multi‐omics strategies could advance our understanding of how specific phenolic compounds influence redox signaling and physiological resilience during physical training.

## Author Contributions

**Kübra Özdemir:** formal analysis (equal), investigation (equal), methodology (equal), writing – original draft (equal), writing – review and editing (equal). **Yeliz Demir:** formal analysis (equal), investigation (equal), methodology (equal), writing – original draft (equal), writing – review and editing (equal).

## Conflicts of Interest

The authors declare no conflicts of interest.

## Data Availability

Data will be made available on request.
